# Reproducibility of up-flow column percolation tests for contaminated soils

**DOI:** 10.1371/journal.pone.0178979

**Published:** 2017-06-05

**Authors:** Tetsuo Yasutaka, Angelica Naka, Hirofumi Sakanakura, Akihiko Kurosawa, Toru Inui, Miyuki Takeo, Seiji Inoba, Yasutaka Watanabe, Takuro Fujikawa, Toshihiko Miura, Shinji Miyaguchi, Kunihide Nakajou, Mitsuhiro Sumikura, Kenichi Ito, Shuichi Tamoto, Takeshi Tatsuhara, Tomoyuki Chida, Kei Hirata, Ken Ohori, Masayuki Someya, Masahiko Katoh, Madoka Umino, Masanori Negishi, Keijiro Ito, Junichi Kojima, Shohei Ogawa

**Affiliations:** 1 Research Institute for Geo-Resources and Environment, National Institute of Advanced Industrial Science and Technology, Tsukuba, Ibaraki, Japan; 2 Center for Material Cycles and Waste Management Research, National Institute for Environmental Studies, Tsukuba, Ibaraki, Japan; 3 Graduate School of Global Environmental Studies, Kyoto University, Sakyo, Kyoto, Japan; 4 Environmental Science Research Laboratory, Central Research Institute of Electric Power Industry, Abiko, Chiba, Japan; 5 Civil Engineering Research Laboratory, Central Research Institute of Electric Power Industry, Abiko, Chiba, Japan; 6 Department of Civil Engineering, Fukuoka University, Jonan, Fukuoka, Japan; 7 Technical Research Institute, Obayashi Corporation, Kiyose, Tokyo, Japan; 8 Core-lab Testing Institute, OYO Corporation, Saitama, Saitama, Japan; 9 Institute of Technology, Shimizu Corporation, Koto, Tokyo, Japan; 10 Center for International Relations, University of Miyazaki, Miyazaki, Miyazaki, Japan; 11 Cold Region Construction Engineering Research Group, Civil Engineering Research Institute for Cold Region, Toyohira, Sapporo, Japan; 12 Pacific Consultants Co.,Ltd, Chiyoda, Tokyo, Japan; 13 Pacific Consultants Environment Research Co.,Ltd, Tsukuba, Ibaraki, Japan; 14 MC Evolve Technologies Corporation, Ami, Ibaraki, Japan; 15 Tokyo Environmental Public Service Corporation, Koto, Tokyo, Japan; 16 School of Agriculture, Meiji University, Kawasaki, Kanagawa, Japan; 17 Taisei Technology Center, Taisei Corporation, Yokohama, Kanagawa, Japan; 18 Kajima Technical Research Institute, Kajima Corporation, Chohu, Tokyo, Japan; 19 Tokai Technology Center, Nagoya, Aichi, Japan; 20 Graduate School of Engineering, Gifu University, Gifu, Gifu, Japan; Leibniz-Institut fur Pflanzengenetik und Kulturpflanzenforschung Gatersleben, GERMANY

## Abstract

Up-flow column percolation tests are used at laboratory scale to assess the leaching behavior of hazardous substance from contaminated soils in a specific condition as a function of time. Monitoring the quality of these test results inter or within laboratory is crucial, especially if used for Environment-related legal policy or for routine testing purposes. We tested three different sandy loam type soils (Soils I, II and III) to determine the reproducibility (variability inter laboratory) of test results and to evaluate the difference in the test results within laboratory. Up-flow column percolation tests were performed following the procedure described in the ISO/TS 21268–3. This procedure consists of percolating solution (calcium chloride 1 mM) from bottom to top at a flow rate of 12 mL/h through softly compacted soil contained in a column of 5 cm diameter and 30 ± 5 cm height. Eluate samples were collected at liquid-to-solid ratio of 0.1, 0.2, 0.5, 1, 2, 5 and 10 L/kg and analyzed for quantification of the target elements (Cu, As, Se, Cl, Ca, F, Mg, DOC and B in this research). For Soil I, 17 institutions in Japan joined this validation test. The up-flow column experiments were conducted in duplicate, after 48 h of equilibration time and at a flow rate of 12 mL/h. Column percolation test results from Soils II and III were used to evaluate the difference in test results from the experiments conducted in duplicate in a single laboratory, after 16 h of equilibration time and at a flow rate of 36 mL/h. Overall results showed good reproducibility (expressed in terms of the coefficient of variation, CV, calculated by dividing the standard deviation by the mean), as the CV was lower than 30% in more than 90% of the test results associated with Soil I. Moreover, low variability (expressed in terms of difference between the two test results divided by the mean) was observed in the test results related to Soils II and III, with a variability lower than 30% in more than 88% of the cases for Soil II and in more than 96% of the cases for Soil III. We also discussed the possible factors that affect the reproducibility and variability in the test results from the up-flow column percolation tests. The low variability inter and within laboratory obtained in this research indicates that the ISO/TS 21268–3 can be successfully upgraded to a fully validated ISO standard.

## Introduction

Environmental impact assessments for both short- and long-term release of chemicals from contaminated soils can be performed through leaching tests. There are several leaching test methods available worldwide, which vary according to factors such as the type of material to be tested, mass, particle size, volume of leachant, addition of leachant (single or renewed), and duration. Leaching tests for contaminated soils can be classified as batch tests [1–6], column tests [7–10]and sequential leaching tests [11–14] or monolithic and bulk tests [15]. Leachability of inorganic substances from contaminated soils depends on several physical parameters such as soil homogeneity, particle size distribution, porosity, hydraulic conductivity, flow rate, contact time between the solid and the solution and temperature, as well as parameters of a chemical nature such as pH value, redox conditions, total organic carbon (TOC) content, chemical speciation of contaminants, complexation reactions and biological activity [16–22].

The column percolation leaching test mimics time-dependent percolation behavior from solids such as contaminated soils in surface waters and groundwater. Even though column percolation tests are performed in laboratories, they resemble natural conditions closer than any other test and provide more robust results than batch tests [17, 23]. Worldwide, several standards for column tests are available: ISO/TS 21268-3(2007), CEN 14405 (2004), NEN 7373 (2004), DIN 19528 (2009), USEPA Method 1314(2013), Nordest Method (1995) and OECD 312 (2004) [7–10, 24–27]. Column tests procedure of ISO/TS 21268–3 are carried out on compacted samples in glass or plastic columns, with an internal diameter of 5 or 10 cm and a height of around 30 ± 5 cm, which are percolated continuously with water or other aqueous solutions such as calcium chloride, usually from bottom to top. Results of concentrations (expressed in mg/L) and cumulative releases (expressed in mg/kg) are plotted in terms of the liquid to solid ratio (L/S) which is the ratio between the total volume of percolated solution (L in litres) and the dry mass of the soil sample (S in kg of dry matter). The ISO-TC190 SC7 WG6, responsible for the development of leaching tests for soil and soil-like materials, has discussed the potential to upgrade ISO/TS 21268–3 to a fully validated standard.

Furthermore, the inter-laboratory reproducibility (difference in test results among laboratories), the within laboratory repeatability (difference in test results within laboratory) are two important components for standardization and reliability of an experimental method. However, a few studies have been conducted to investigate repeatability and reproducibility of column tests for contaminated soils [28, 29]. Garrabrants et al. (2012) conducted interlaboratory validation of the USEPA Method 1314 (US EPA, 2013) up flow percolation test [29]. They concluded that Method 1314 showed good repeatability and reproducibility, with a mean reproducibility of 24% RSD_R_ (reproducibility relative standard deviation) for eluate concentration and 16% RSD_R_ for cumulative mass release. The repeatability values were 6% RSD_r_ (repeatability relative standard deviation) for eluate concentration and 16% RSD_r_ for cumulative mass release. They also pointed out that when the Method 1314 was performed in different laboratories using homogenized samples of the same material, the variation in test results would be expected to be less than 30%.

Kalbe et al. (2008) evaluated the cumulative amount of PAH for one contaminated soil at six different laboratories following ISO/TS 21268–3. Good reproducibility was found, but the evaluation did not take inorganic substances into consideration, nor was statistical analysis conducted [17]. Geurts et al. (2016) evaluated the average cumulative release of inorganic substances at L/S 10 L/kg for two soils and four sediments and showed good repeatability and reproducibility [28]. However, only the average cumulative release at L/S 10 L/kg was evaluated; in ISO/TS 21268–3, seven fractions of L/S (0.1, 0.2 0.5, 1, 2, 5, 10 L/kg) are required. Therefore, further validation studies are necessary in order to upgrade ISO/TS21268-3 to a standard method.

This study aims to evaluate the reproducibility of up-flow column percolation tests conducted in Japan with participation of 17 research institutes, universities and private enterprises around the country and furtherly investigate the difference between duplicated results obtained within one laboratory. Column percolation tests were performed following the technical specification ISO/TS 21268–3 considering the leachability of anions and cations. Our presented results can be used in the future as support for upgrading this technical specification to a fully validated ISO.

## Materials and methods

### Materials

Three different soils, hereafter referred as to Soils I, II and III, were used to perform column test experiments. Soil I was a naturally contaminated soil, whereas Soils II and III were anthropogenically-contaminated soils. All soils used in this research were collected in the field and not spiked with contaminants, sieved and homogenized in the laboratory, distributed to participating laboratories (in case of Soil I). Soils II and III correspond to Soils B and D reported in [16]. Column tests with Soils II and III were performed again for the purpose of this research. Table 1 shows the physical and chemical characteristics of the soils. The maximum particle size and moisture content were measured according to JIS A 1203 (2009) [30], loss of ignition according to JIS A 1226 (2000) [31], particle density according to JIS 1202 (2009) [32], particle size distribution according to JIS A 1204 (2009) [33], and total element content according to the methods used for bottom sediment [34]. Following the Basic System of Soil Classification (Soil Textural Triangle) developed by the United States Department of Agriculture (USDA) system, Soils I, II and III correspond to sandy loam.

**Table 1:** Properties of all soils.

Parameter		Dimension	Soil I	Soil II	Soil III
Maximum particle size		mm	4.75	2	2
Average moisture content		% by mass	21.6	10.9	26.6
Loss of ignition		% by mass	6.0	3.7	9.5
Cation exchange capacity		cmol_c_/kg	8.1	8.5	10.3
Particle density		g/cm^3^	2.683	2.742	2.740
Particle size distribution	2–4.75 mm	% by mass	7.5	-	-
	0.850–2 mm	% by mass	9.2	77.8	54.4
	0.250–0.850 mm	% by mass	22.2		
	0.075–0.250 mm	% by mass	25.6	10.1	34.3
	0.005–0.075 mm	% by mass	18.3		
	< 0.005 mm	% by mass	17.2	12.1	11.3
Total elemental content	Cu	mg/kg	33.1	NA	NA
	As	mg/kg	12.0	745.9	NA
	Ca	g/kg	32.0	NA	NA
	F	mg/kg	140.7	4108.7	NA
	Pb	mg/kg	NA	1262.2	NA
	Cd	mg/kg	NA	4.3	NA
	Cr	mg/kg	NA	43.4	NA
	Se	mg/kg	NA	32.8	NA

NA: not available, Soils II and III are the same as Soils B and D in research [16]

Approximately 40 kg of Soil I was brought from the field and sieved using a 4.75-mm opening mesh. The soil was divided into smaller portions using the coning and quartering method and weight of each portion was approximately 1 kg. Two of them were distributed to each participating laboratory as test specimens. Likewise, approximately 10 kg of filed-collected Soil II or Soil III was divided into smaller potions with 1 kg in each portion.

### Concept

Leaching of chemicals from three different soils were evaluated by column percolation tests at two different equilibration times (16 h and 48 h) and flow rates (12 and 36 mL/h). The experimental conditions are summarized in Table 2. For Soil I, all column experiments were conducted following the technical specification ISO/TS 21268–3 [7] with 48 h of equilibration time and a flow rate of 12 mL/h. Seventeen institutions performed experiments with Soil I, with each laboratory conducting column tests in duplicate, allowing the evaluation of both reproducibility and variability in results within laboratory. As every test was performed in duplicate, we do not use the term “repeatability”, but the term “difference in results within laboratory”. Column tests with Soils II and III applied 16 h of equilibration time and a flow rate of 36 mL/h, following the method proposed by Naka et al. [16]. Tests performed with Soils II and III were conducted by one laboratory, in duplicate to allow the evaluation of the variability in column test results.

**Table 2:** Experimental conditions.

Parameter	Unit	Soil I	Soil II	Soil III
Experimental period	-	January to April 2015	June 2016	May 2016
Number of laboratories	-	17 (N = 2)	1	1
Number of experiments	-	34	2	2
Sample	-	Naturally-contaminated sandy soil	Anthropogenically-contaminated sandy soil	Anthropogenically-contaminated sandy soil
Sampling (cumulative L/S)	L/kg	0.1, 0.2, 0.5, 1, 2, 5 and 10	0.1, 0.2, 0.5, 1, 2, 5 and 10	0.1, 0.2, 0.5, 1, 2, 5 and 10
Column diameter	cm	5	5	5
Column height	cm	30	30	30
Sample state	-	Wet, natural water content	Wet, natural water content	Wet, natural water content
Packing method	-	15 layers using a 125g rammer	15 layers using a 125g rammer	15 layers using a 125g rammer
Eluant	-	Deionized water with 0.001 M CaCl_2_	Deionized water with 0.001 M CaCl_2_	Deionized water with 0.001 M CaCl_2_
Measured parameters	-	pH, EC, Cu, As, Se, Cl, Ca and F	pH, EC, Se, As, Cu, Mg, F and DOC	pH, EC, Se, As, Cu and B
Reproducibility	-	Evaluated	NA	NA
Difference in results within laboratory	-	Evaluated	Evaluated	Evaluated

## Methods

**Fig 1:**
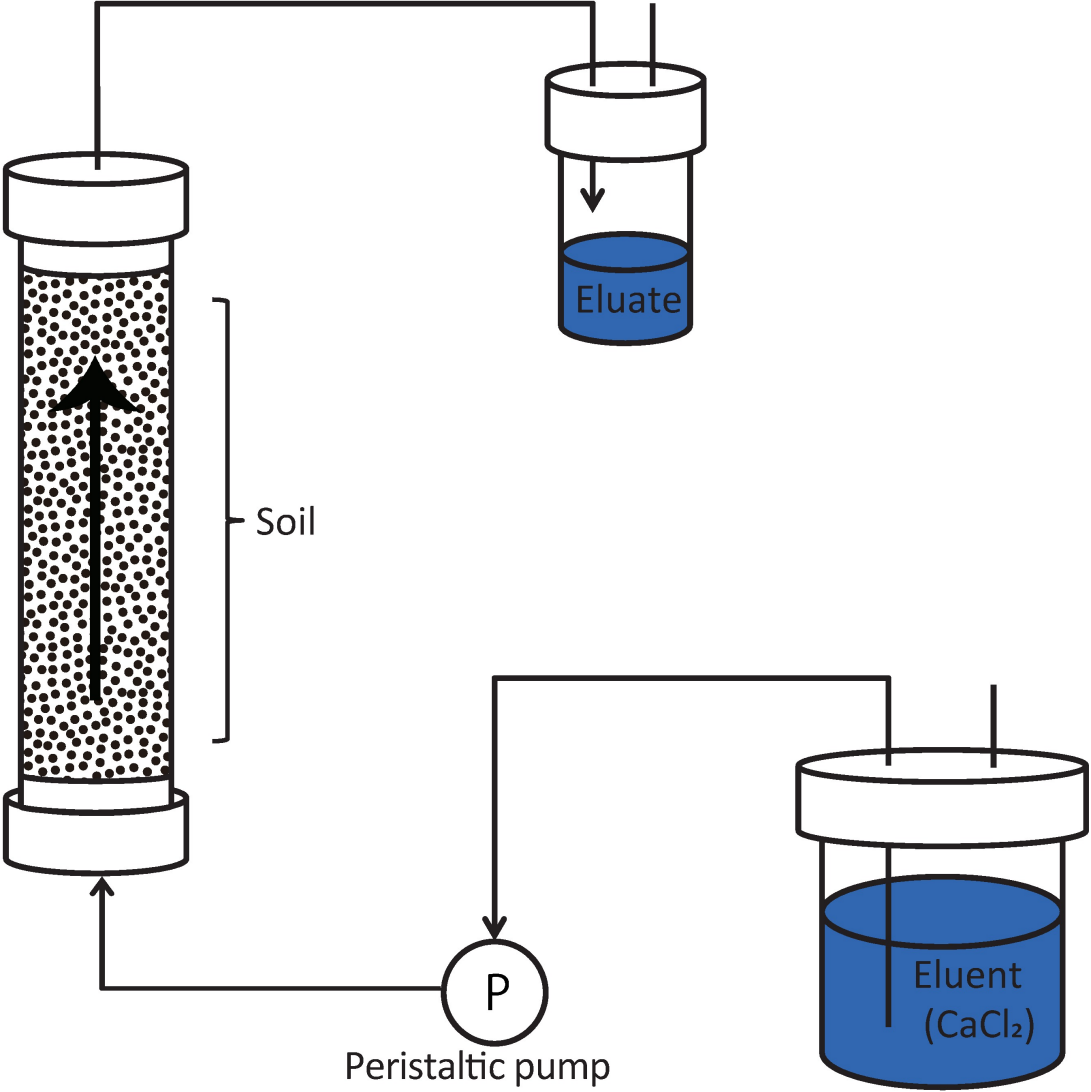
Diagram of the up-flow column percolation test (Modified from Naka et al., [16]).

The up-flow column percolation tests were conducted following the procedure described in ISO/TS 21268–3 [7]. A set up of the method is presented in Fig 1.

Five research institutes, four universities, four construction companies and four consulting companies (analysis companies) located throughout Japan participated in this validation test using Soil I. Each institute performed blank tests (without soil) and two column tests, which were carried out from January to April 2015. Additionally, one laboratory was selected to perform experiments in duplicate with both Soils II and III to determine the difference in results within laboratory.

The specimen was packed into the column with a modification (i.e. the specimen was not dried in the laboratory). Approximately the same amount of soil was prepared and divided it equally into 15 small portion carefully weighed. It was filled into a column, up to a bed height of 30 cm in five layers and each layer were introduced into the column in three sub-layers. Each layer was packed using a rammer with a weight of 125 g and the rammer dropped three times onto each layer from 20 cm height [7]. Approximately the same amount of soil was used in each test. To prevent soil material loss and facilitate uniform distribution of eluent, a filter paper and a 3-mm high plastic plate with several holes were placed at the bottom of the column before packing the column with soil. The column was packed by filling it with the soil specimen up to a height of 30 ± 5 cm in five layers After the final sub-layer was packed, a plastic plate and a filter paper were placed on the top section of the column.

We connected Tygon tubes (inner diameter of 0.8 to 6 mm, depending on the design of the column) to the bottom and top of the columns. We connected the bottom (inlet) to a tank containing 0.001 M CaCl_2_ (eluent) and the top (outlet) to an eluate collection bottle of an appropriate size (plastic bottles with volumes ranging from 100 mL to 4 L). We prepared the eluent solution as 0.001 M CaCl_2_ using high purity CaCl_2_ and de-ionized water. The system was equipped with a peristaltic pump to allow the eluent to pass through the column (from the bottom to the top) at a constant flow rate of 12 mL/h.

Once the system was set, we allowed the eluent to percolate through the specimen until it reached the top of the column. At this point, we stopped the peristaltic pump and allowed the system to equilibrate for 48 h. The fractions collected were 0.1 ± 0.02, 0.2 ± 0.04, 0.5 ± 0.08, 1 ± 0.15, 2 ± 0.3, 5 ± 0.4, and 10 ± 1 L/kg of dry mass. As soon as the samples were collected, we recorded the weight, then removed a small portion of the sample for pH and electrical conductivity (EC) analysis. We filtered the eluate afterwards using a 0.45-μm pore size membrane filter, then separated it into two aliquots: one preserved with 0.5% concentrated nitric acid for cation analysis, and the other remained unpreserved for anion concentration analysis.

We analyzed all elute in one laboratory. We measured Cu, As, Se, Ca and Mg concentrations with inductively coupled plasma-optical emission spectrometry (Vista-PRO Simultaneous ICP-OES, SII and Varian, Agilent Technologies, Santa Clara, California, USA) and inductively coupled plasma-mass spectrometry (ICP-MS 8800 Series, Agilent Technologies, Santa Clara, California, USA), and measured B, Cl and F by ion chromatography (Dionex ICS-2000 ion chromatograph operating, Thermo Scientific, Sunnyvale, California, USA). We measured dissolved organic carbon (DOC) with a TOC analyzer (Shimadzu TOC-VCPH, Shimadzu Corp., Kyoto, Japan).

### Statistical analysis

Reproducibility and repeatability are standardized terms associated with the precision of measurements on the same specimen using the same test method under certain experimental conditions [35–37]. The measure of the variation as a result of different laboratories performing the same test method on the same material is defined as the reproducibility of measurements. The variation in results from experiments conducted within each laboratory by the same operator and the same equipment on the same material is the repeatability of measurements.

The reproducibility of column percolation tests for Soil I was determined in terms of the coefficient of variation (CV). The CV is a measure of the variability relative to the mean. It is calculated by dividing the standard deviations of “inter laboratory” or “within laboratory” results by the mean and multiplying the result by 100 and is presented as a percentage.

Considering that column tests with Soils I, II and III were conducted in duplicate within one laboratory, we do not used the term “repeatability”, but instead, the variability within laboratory was reported in terms of the difference between results obtained by each laboratory, divided by their mean and multiplied by 100 and is presented as a percentage.

## Results

### Sample conditions of Soil I

The water content (expressed in % by mass), dry weight (in g) and height (in cm) of the specimen reported for every column test are presented in S1–S3 Figs. The average water content of the specimen was 21.6 ± 0.8%, the average dry weight was 662 ± 28 g and the average height was 30.7 ± 1.7 cm. In addition to water content and dry weight, each laboratory reported the flow rate (S4 Fig) and the ratio between the measured and target cumulative L/S (S5 Fig) for each column experiment. The criteria applied in this research to eliminate outliers was by discarding observation points in which flow rate values were outside the range 15 ± 2 cm/day or 12.3 ± 1.6 mL/h (range stipulated by the ISO/TS 21268–3) and observation points for which the ratio between measured and target cumulative L/S was lower than 0.8 or greater than 1.2. Fifteen data points were eliminated at this stage.

High concentrations of Cu were identified in two blank column tests, both from the same institution. High values of Cu concentration in blank tests can be attributed to contamination from previous samples, thus, all Cu results for this institution were eliminated from the database. Moreover, two Se observation points, one laboratory at cumulative L/S = 0.1 L/kg and another laboratory at cumulative L/S = 5 L/kg were also discarded because they were the only data with z-score values greater than 5 (outliers). The z-score indicates how many standard deviations a data point is from the mean. For example, a z-score equal to 1 that an element concentration is 1 standard deviation greater than the mean concentration of this element In this study, data with z-score less than -3 or greater than 3 were considered outliers. The effective data after eliminating all outliers are shown in S1 Table.

### Concentration and cumulative amount of Soil I

**Fig 2:**
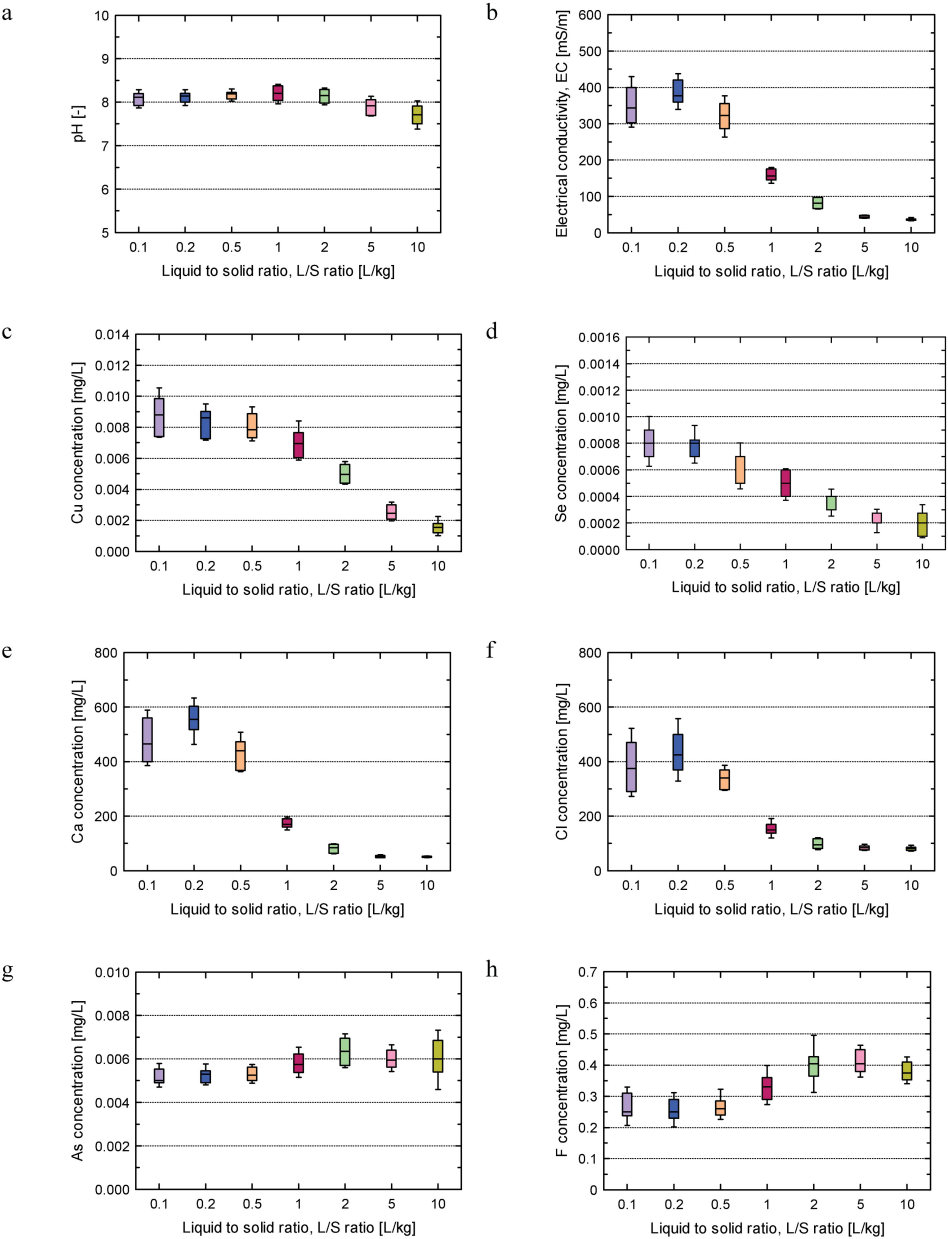
Boxplot of parameters: pH, EC, and concentrations of Cu, As, Se, Cl, Ca and F for Soil I. Whiskers represent mean ± standard deviation values.

**Fig 3:**
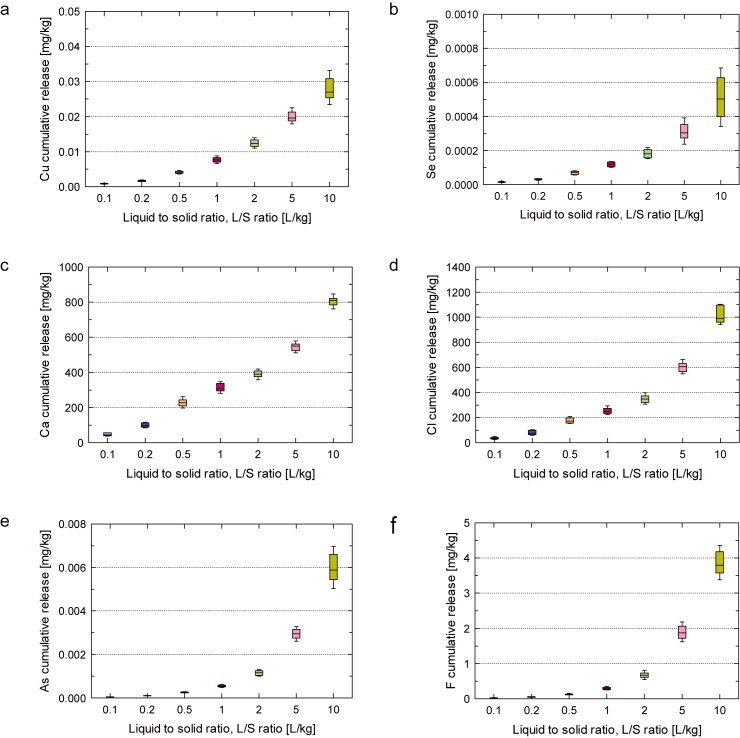
Boxplot of cumulative releases of Cu, As, Se, Cl, Ca and F for soil I. Whiskers represent mean ± standard deviation values.

Boxplots of the effective results of pH, electrical conductivity (EC) and concentrations of Cu, As, Se, Cl, Ca are presented in Fig 2. Boxplots of the cumulative releases of Cu, As, Se, Cl, Ca and F are shown in Fig 3.

Fig 2 shows that the pH was almost constant, ranging from 7.5 to 8.5 approximately throughout the duration of the test, which indicates that the soil was stable at the conditions set for column experiments. The EC (ranging from 50 to 450 mS/m approximately) and the concentrations of Cu (ranging from 0.001 to 0.015 mg/L approximately) and Se (ranging from 0.0001 to 0.001 mg/L approximately) gradually decreased over time. The concentrations of Ca (ranging from 50 to 600 mg/L approximately) and Cl (ranging from 100 to 500 mg/L approximately) presented the same pattern, with an initial increase in concentration, followed by a rapid decrease in concentration. No large fluctuations were apparent in the As concentration over time (ranging from 0.005 to 0.007 mg/L approximately), indicating that the release of As from Soil I was constant over time (Fig 2G). In contrast, the F concentration (ranging from 0.2 to 0.5 mg/L approximately) showed a complicated pattern with gradual decrease initially, followed by a subsequent a rapid increase, and a constant concentration at L/S values > 5 L/kg (Fig 2H).

Whiskers presented in Fig 3 show one standard deviation above and below the mean of the data. Fig 3 shows that cumulative releases of Cu (Fig 3A), Se (Fig 3B), Ca (Fig 3C), Cl (Fig 3C), As (Fig 3D) and F (Fig 3E) present good reproducibility up to L/S ratio of 5 L/kg. However, greater variation was observed at L/S 10 L/kg, except for Ca concentration.

### Reproducibility of the up-flow percolation test of Soil I

**Fig 4:**
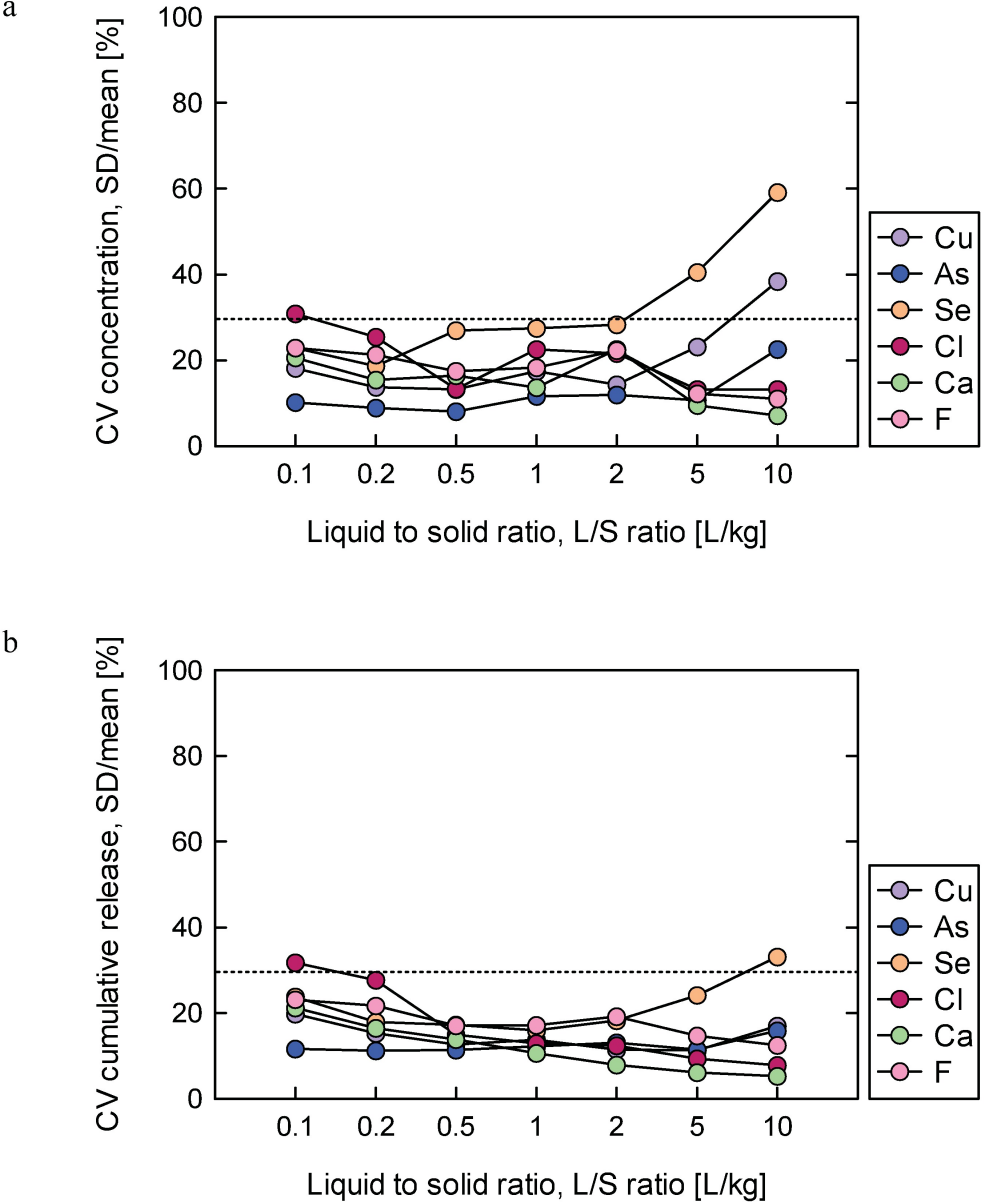
Coefficient of variation for Cu, As, Se, Cl, Ca and F (a) concentrations and (b) cumulative releases for Soil I.

To quantitatively determine the reproducibility of the validation study results, the CV was calculated for concentrations of Cu, Se, Ca, Cl, As and F for all available data (Fig 4A) and cumulative releases of the same elements (Fig 4B) for Soil I.

Results presented in Fig 4A show that the CV of more than 90% of the data was below 30%, which indicates good reproducibility. A CV higher than 30% was observed for Cl at L/S = 0.1 and for Cu and Se at L/S > 5 L/kg. Fig 3 shows that the CV of more than 95% of the cumulative concentration data were below 30%, with the majority lower than 20%.

### Difference between results within laboratory for Soils I, II and III

**Fig 5:**
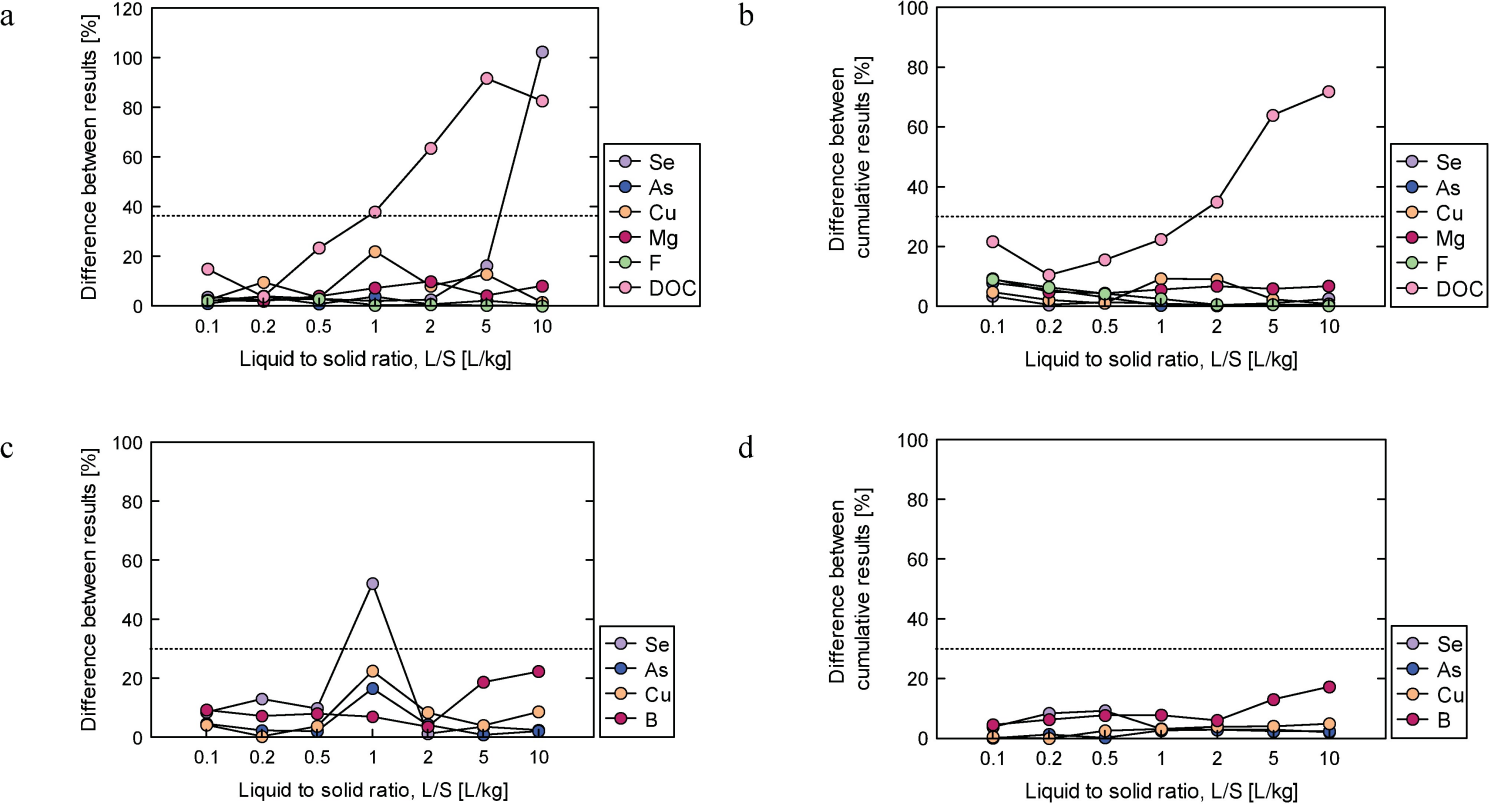
Difference in results within laboratory for Cu, Se, As and F concentration for Soil I. The “difference within laboratory” corresponds to the difference between the results obtained in the same laboratory, divided by their mean and expressed in terms of percentage.

Fig 5 and S6 Fig show that the difference between results within laboratory obtained by each of the 17 laboratories that examined Soil I. These results indicate that the differences within laboratory were lower than 30% in 89% of the Cu data, 75% of the Se data, 99% of the As data and 92% of the F data.

**Fig 6:**
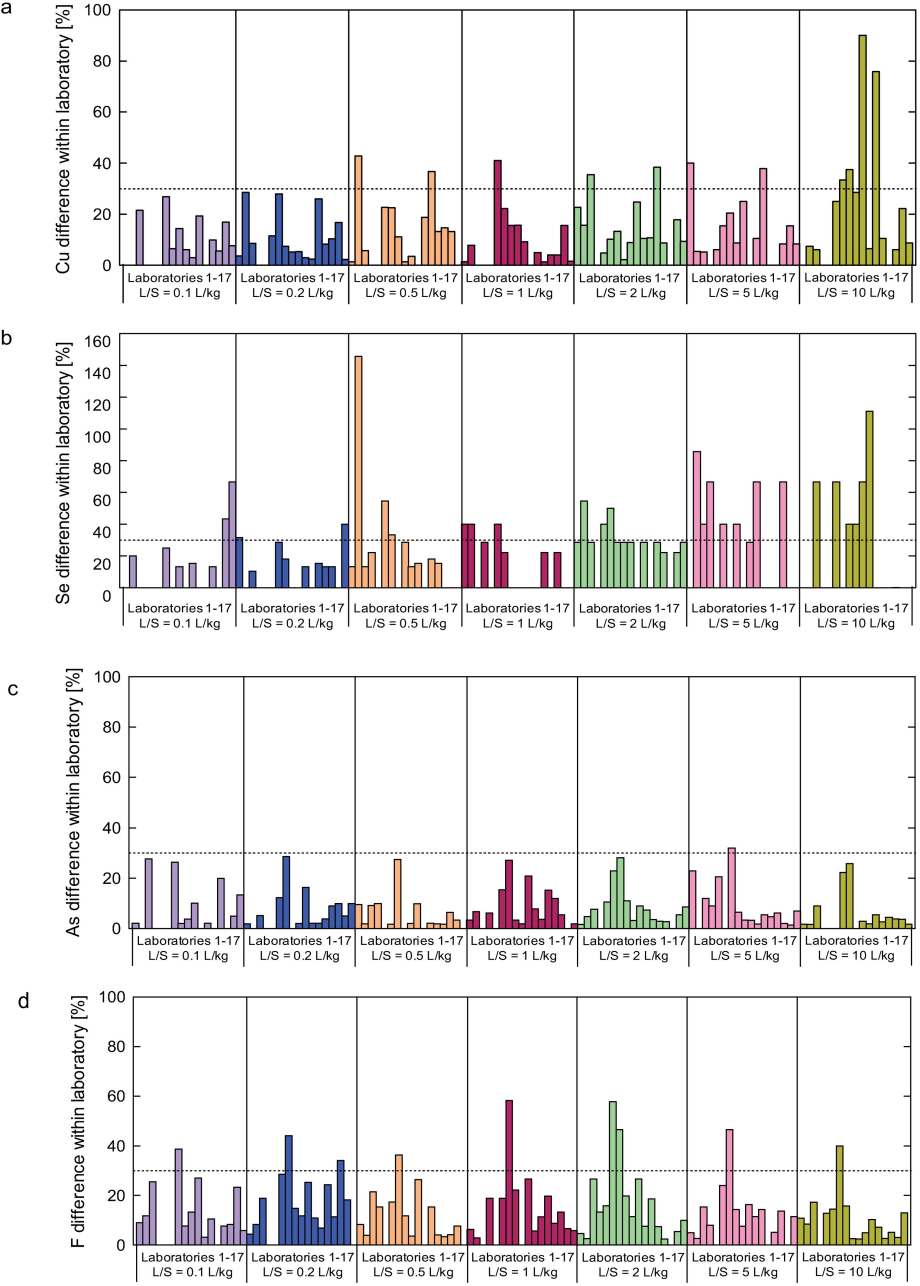
Difference in results within laboratory for Se, As, Cu, Mg, F and DOC (a) concentrations and (b) cumulative releases for Soil II; difference in results within laboratory for Se, As, Cu and B (c) concentrations and (d) cumulative releases for Soil III. The “difference between results” and “difference between cumulative results” corresponds to the difference between concentrations and cumulative releases, divided by their mean and expressed in terms of percentage.

In this research project, each laboratory conducted only two column tests. To obtain a better judgement of the difference in results within laboratory, extra column tests were performed with two different soils (Soils II and III). Column experiments were also conducted in duplicate and the difference between results of both concentrations and cumulative releases were determined. The differences between Soil II results are presented in Fig 6A (concentration) and Fig 6B (cumulative releases). The corresponding results of concentrations and cumulative releases for Soil III are presented in Fig 6C and 6D, respectively. The Soil II results show that the difference between values was lower than 30% for more than 88% of the concentration data and 93% for cumulative release data. Results obtained for Soil III indicate that 96% of the difference between concentration data were lower than 30% and 100% of the cumulative release data were below 30% (Fig 6C and 6D).

## Discussion

### Challenges of reproducibility in terms of test procedure: From filling to sampling

Validation studies face several challenges in terms of the test procedure, from the filling stage to sampling. These challenges confirm whether the current test conditions as required by ISO TS 21268–3, which include the filling method, percolating solution or eluent, pump type, eluent flow rate and sampling timing, are sufficient for upgrading the ISO/TS 21268–3 to a fully validated ISO standard from the view of reproducibility. Results obtained in this research showed that there was no great difference in either the weight (the average dry weight was 662 ± 28 g, S2 Fig) or the height (30.7 ± 1.7 cm, S3 Fig) of the soil in the columns. This suggests that all participants successfully followed the filling method. Some institutions conducted column tests with different brands of peristaltic pumps, but this did not represent any problem because participants adjusted the flow rate before starting the tests. Since the flow rate was carefully adjusted, few participants faced difficulties in keeping the flow rate constant at the beginning of the tests and/or through the duration of the test. The sampling time represents a big challenge, especially when sampling has to be conducted during the night. According to the ISO method, seven samples must be collected at cumulative L/S ratios of 0.1, 0.2, 0.5, 1, 2, 5 and 10 L/kg. Thus, if sampling is not carefully scheduled or a delay occurs as a result of experimental troubles, there is the possibility that sampling has to be performed at night, making percolation column tests less practical.

### Reproducibility results

We evaluated reproducibility using Soil I data (Fig 4). For more than 90% of the data for Soil I, the CV was below 30% and 80% of the CV value was below 25%. The mean reproducibility was 20% for concentration and 15% for cumulative release.

Moreover, we compared reproducibility Cv obtained in this study with previous research results. Garrabrants et al. (2012) conducted inter-laboratory validation of the USEPA Method 1314 which is an up flow percolation test and obtained a mean reproducibility of 24% RSD_r_ for eluate concentration and 16% RSD_r_ for cumulative mass release [29]. In this research, all sample were analyzed in one laboratory, which is the same as in our study. They pointed out that when different laboratories perform tests following the Method 1314 on homogenized samples of the same material, the variation in test results is expected to be less than 30%.

Geurts et al. (2016) evaluated the average cumulative release of inorganic substances at L/S 10 L/kg for two soils and reported average RSD values of 15% (ranging from 6 to 42%) for contaminated soil and 44% (ranging from 11 to 90%) for sieved sand at cumulative release (L/S = 10 L/kg). The RSD values of cumulative release of up flow percolation test applied to sediment were 25–50% relative high compared with contaminated soil[28]. Furthermore, The reproducibility of Toxicity Characterization Leaching Procedure (TCLP) which is one of the famous methods for characterizing the hazardous material, was evaluated using three waste and obtained reproducibility were a mean RSD of 74% [38]. Our reproducibility values in terms of CV (%) for both concentration and cumulative release (Fig 4B) obtained in this study were lower than or equal to their results.

Comparative study of reproducibility results is sufficient for a conclusion that reproducibility of ISO/TS 21268–3 is "good" from not only the cumulative release at L/S 10 evaluated by Geurts et al. (2016) but also all seven concentration and cumulative releases of L/S 0.1, 0.2 0.5, 1, 2, 5, 10 L/kg for inorganic constitutes in this study, indicating that the technical specification ISO/TS 21268–3 can be upgraded to a fully validated ISO standard for inorganic constituents.

However, it is important to mention that low reproducibility results with CV values greater than 30% or around 20% were observed for some substances at certain L/S values. The reasons for the low reproducibility in these cases are considered in the following paragraphs.

### Effect of a low concentration range on reproducibility

It is well known that the concentration decreases with increasing the CV value [39, 40]. As shown in Fig 4, Se leaching reproducibility was low, and further decreased as the L/S value increased. These results were due to the large analytical errors caused by the low Se concentrations. The leaching of Se took place at in the early stages of the column percolation test, leading to a very low Se concentration (below 1 ppm, Fig 2D) at higher L/S values. In other words, as the concentration decreased, the margin of error increased due to the ratio of data to “error noise” decreasing; small errors will have a greater impact on low concentration measurements, thus impacting the reproducibility.

In addition, the reproducibility for Cu at L/S = 10 L/kg was also low. Similar to Se, low concentrations of Cu lead to subsequent analytical errors (Fig 2C). Thus, a low concentration of a leached substance may lead to low reproducibility.

### Effect of substances with high initial leaching concentration at low L/S values on reproducibility

We found that CV values of Ca and Cl, easily leached at L/S ≤ 0.5, were high at L/S = 0.1 and 0.2, and then gradually decreased as the L/S value increased (Fig 4). This low reproducibility may be related to the easy leaching characteristics of Ca and Cl at L/S ≤ 0.5 L/kg. Unlike Cu and Se, which is low concentration and gradually decreased in leaching concentration, Ca and Cl showed high initial leaching concentrations because Ca and Cl exist mainly adsorbed form by the charge of soil particle surface and exchange easily. For such substances, the difference in initial saturation time, initial flow rate, or L/S actually sampled in the range would affect their initial leaching concentrations. Such a pattern of decrease in leaching concentration may cause low reproducibility at low L/S values for the substances with high initial leaching concentrations.

### Effect of the proximity of changes to leaching concentration within tests on reproducibility

The F leaching concentration showed a complex behavior compared with other substances, decreasing at L/S ≤ 0.5 L/kg, then increasing at near L/S = 1 L/kg, then finally stabilizing at near L/S = 5 L/kg. Fig 3 shows CV values with around 20% for F at L/S ≤ 2 L/kg. The low reproducibility of F can be attributed to this complex behavior.

And As showed stable leaching concentrations. Along with the finding of high reproducibility with CV 10% for As (at L/S ≤ 5 L/kg) exhibiting very little change in leaching concentration, low reproducibility may occur in the proximity of changes to leaching concentration within tests (e.g. F leaching concentration initially increasing, but then decreasing after a certain point in the test).

### Difference between results conducted within laboratory

Good repeatability results were obtained for Soil I. For instance, the leaching concentration of As from all laboratories was 10 ppb throughout the duration of the test and the CV, indicating the degree of reproducibility, was lower than 30%, except for one data point (Fig 5 and S6 Fig). However, there were some cases in which the difference between results was over 30%. The reasons for this are considered in the following paragraphs.

Great differences between results within laboratory were found in all Se cases. The difference within laboratory results, as well as the Se leaching concentration, gradually decreased as the L/S value increased. In addition, the low difference between values for Cu at L/S = 10 was also due to its low concentration (lower than or equal to 1 ppb). This indicates that a low leaching concentration of the target substance may lead not only to low reproducibility but also to great difference between values.

A difference of over 30% in the F leaching concentrations was observed in many cases. As stated for reproducibility, this low repeatability may be related to a complex behavior of F in the column, when compared to other substances. It should be noted that a low repeatability may occur in the proximity of changes to leaching concentration within tests (e.g. the leaching concentration of F initially increased, but then decreased after a certain point).

## Conclusions

The validation studies of up-flow column percolation tests following ISO/TS 21268–3 were conducted in duplicate by 17 institutions in Japan for Soil I. Column tests for Soils II and III were conducted in a single laboratory. Good reproducibility was obtained for Soil I and high repeatability was obtained for Soils I, II and III.

The reproducibility was measured in terms of the CV. For more than 90% of the data for Soil I, the coefficient of variation was below 30%, which indicates good reproducibility. Tests to quantify the repeatability showed that CV values for 75–95% of the data were below 30% for Soil I. Low reproducibility was observed for low concentration ranges, for substances with high initial leaching concentration at low L/S values and in the proximity of changes to leaching concentration within tests.

The difference in results within laboratory was 30% or less for more than 93%, 88% and 96% of the data for Soils I, II, III, respectively. In order to obtain more accurate data, we suggest conducting further column experiments with triplicated tests as a minimum,; in this study, each laboratory performed only duplicate tests.

The overall results suggest that the technical specification ISO/TS 21268–3 can be upgraded to a fully validated ISO standard.

## Supporting information

S1 Fig Water content of Soil I for each column experiment. (TIF)

S2 Fig Dry weight of Soil I reported for each column experiment. (TIF)

S3 Fig Height of Soil I reported for each column experiment. (TIF)

S4 Fig Flow rate reported for each column experiment and for every cumulative liquid-to-solid ratio for Soil I. (TIF)

S5 Fig Ratio between measured and target liquid-to-solid ratio for each column experiment for Soil I. (TIF)

S6 Fig Difference in results within laboratory for Cu, Se, Ca, Cl, As and F cumulative releases for Soil I. The “difference within laboratory” corresponds to the difference between cumulative releases obtained in the same laboratory, divided by their mean and expressed in terms of percentage.
(TIF)

S1 Table Effective data obtained from interlaboratory column tests for Soil I. (DOCX)
