# Supplementary figures and images for: Reproducibility of up-flow column percolation tests for contaminated soils
Source: PLoS One. 2017 Jun 5;12(6):e0178979. doi: 10.1371/journal.pone.0178979 (PMC5459554; doi:10.1371/journal.pone.0178979)

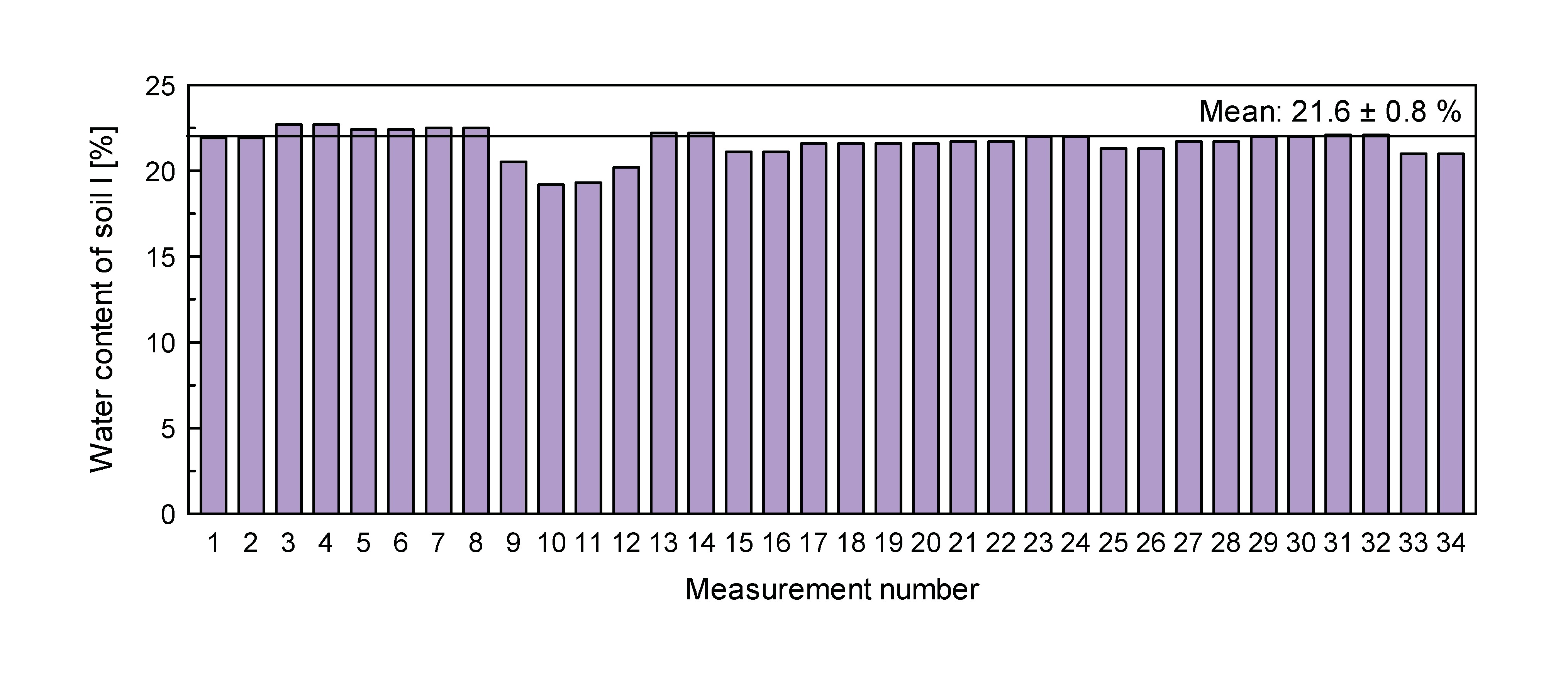

Supplement: S1 Fig — (TIF) [file pone.0178979.s001.tif]

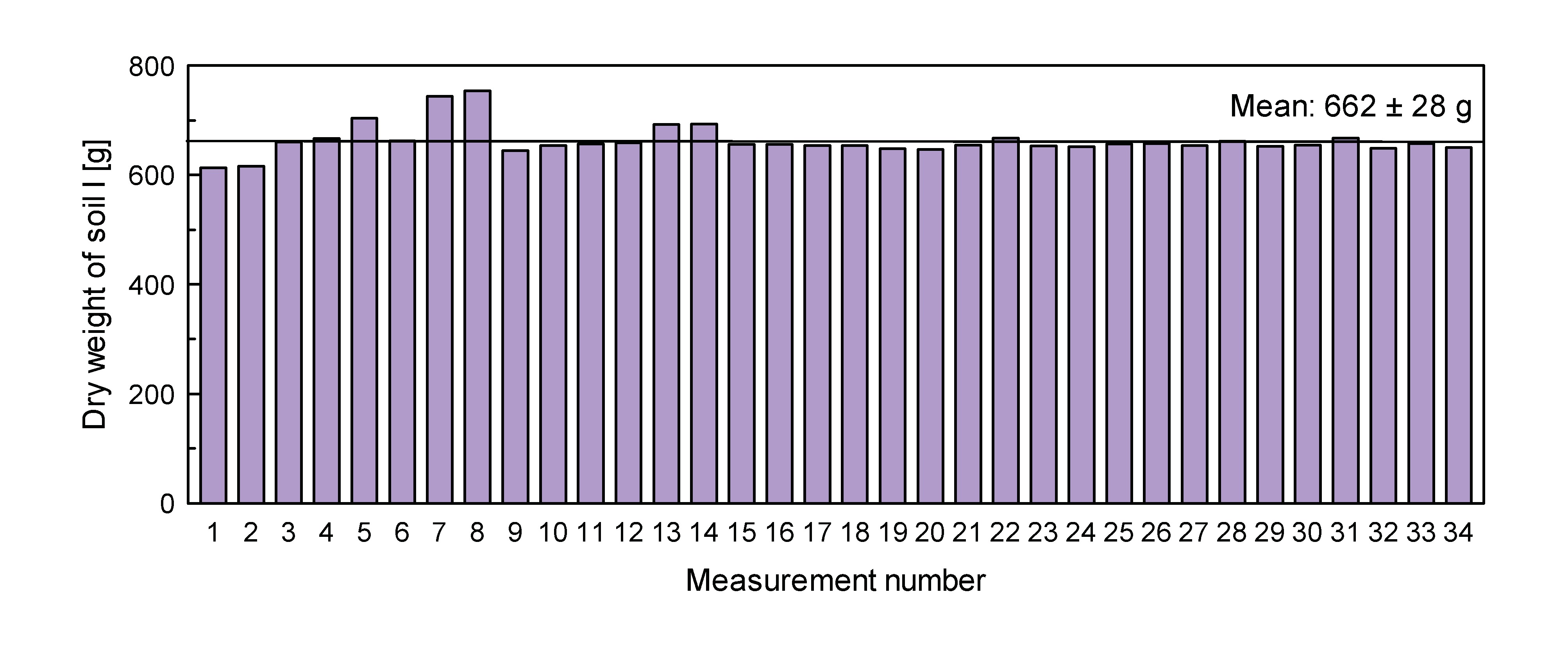

Supplement: S2 Fig — (TIF) [file pone.0178979.s002.tif]

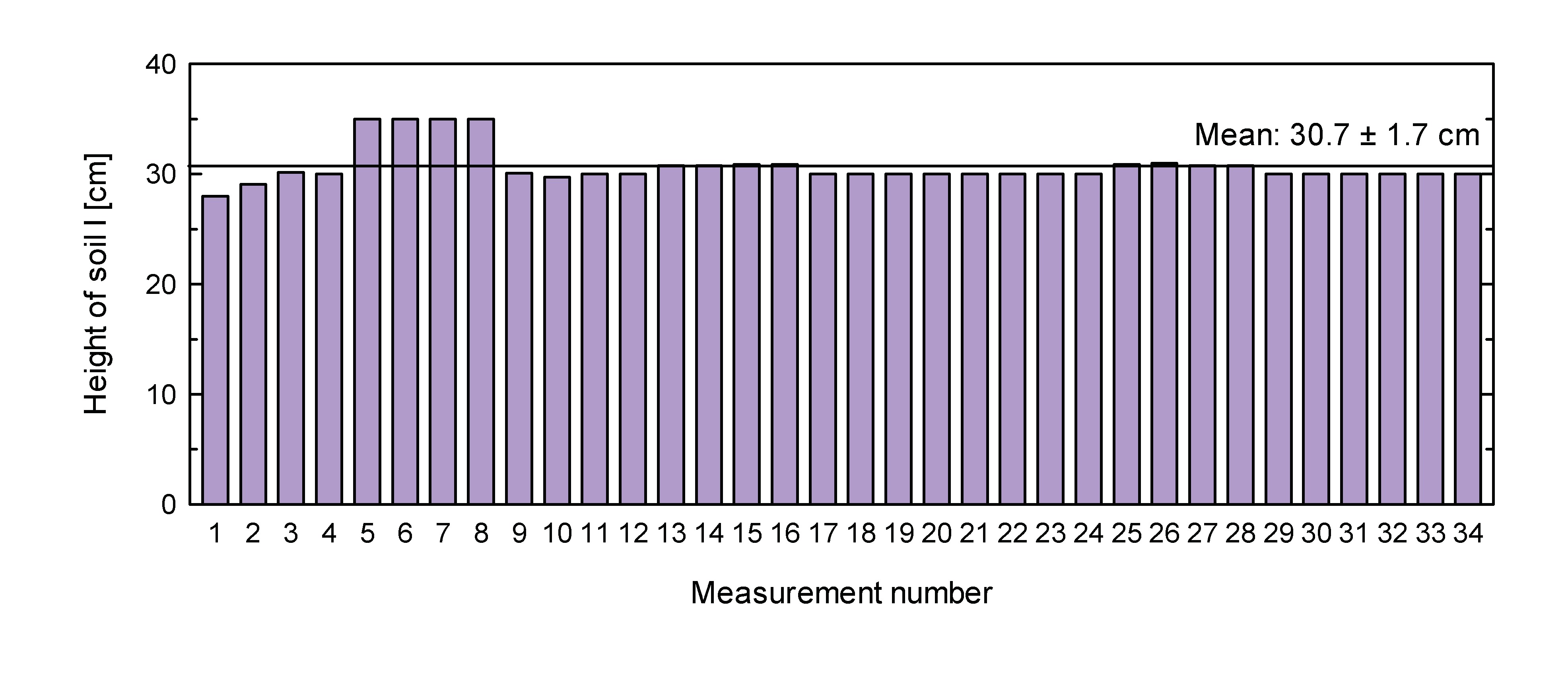

Supplement: S3 Fig — (TIF) [file pone.0178979.s003.tif]

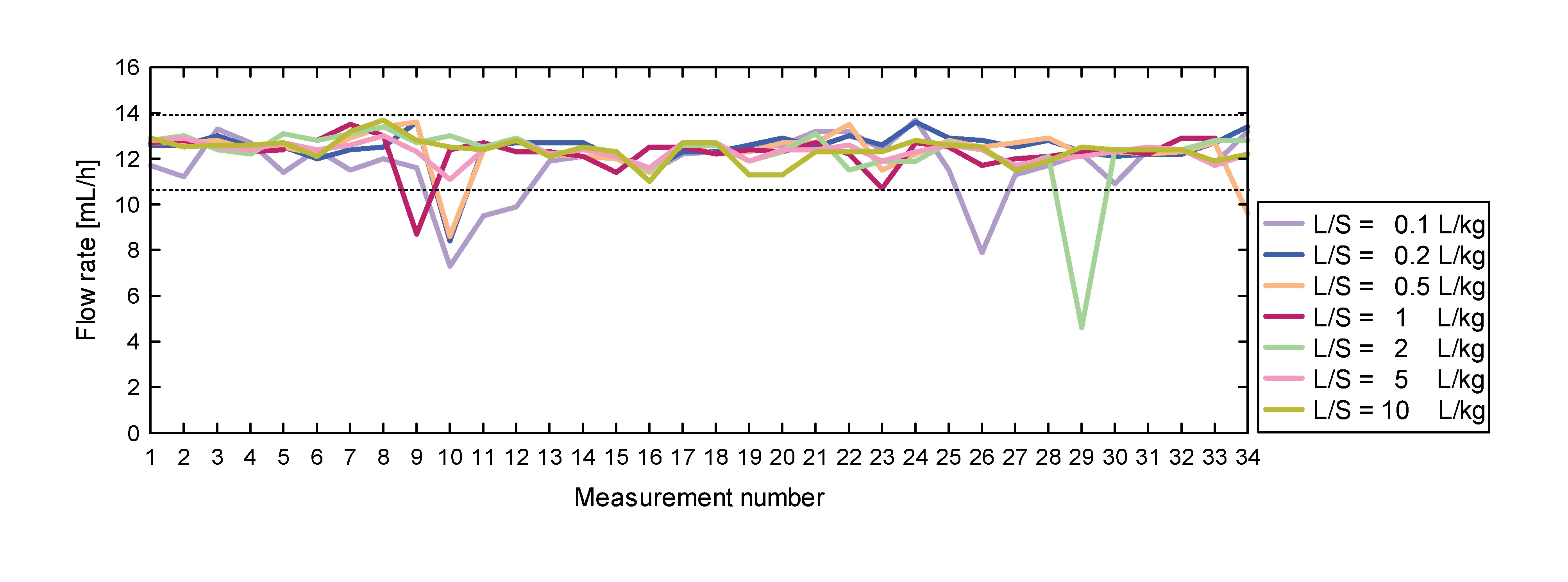

Supplement: S4 Fig — (TIF) [file pone.0178979.s004.tif]

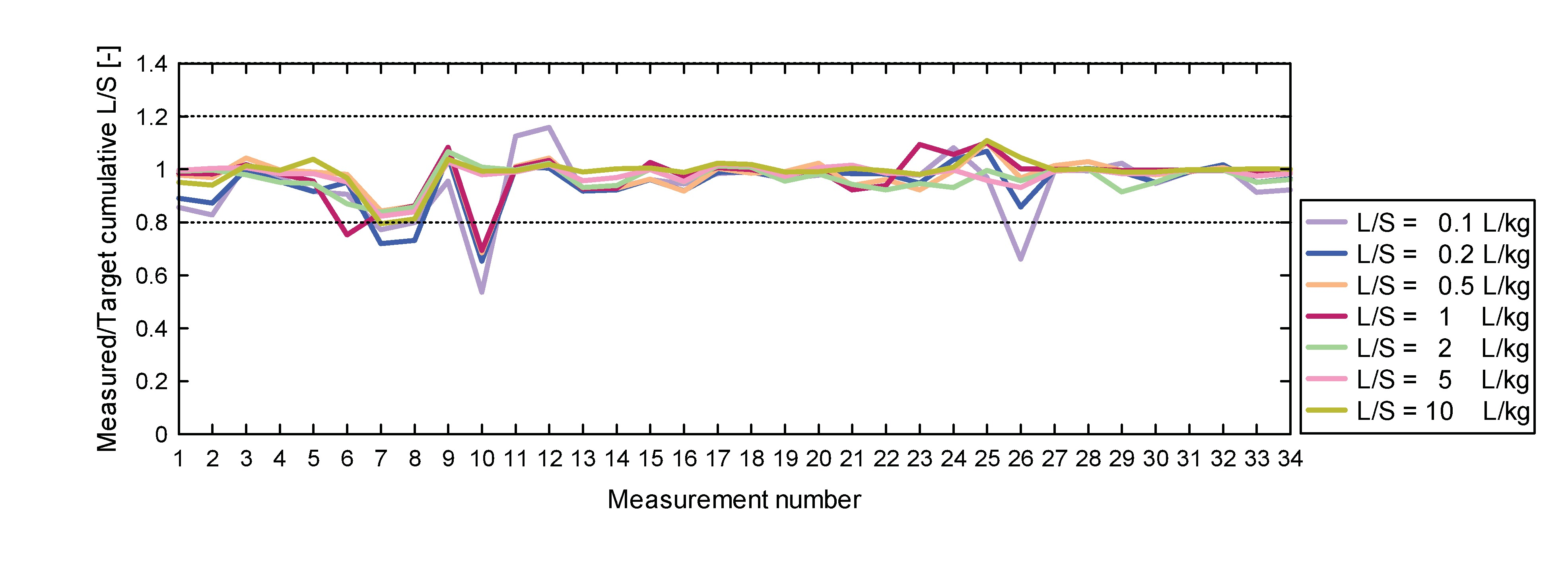

Supplement: S5 Fig — (TIF) [file pone.0178979.s005.tif]

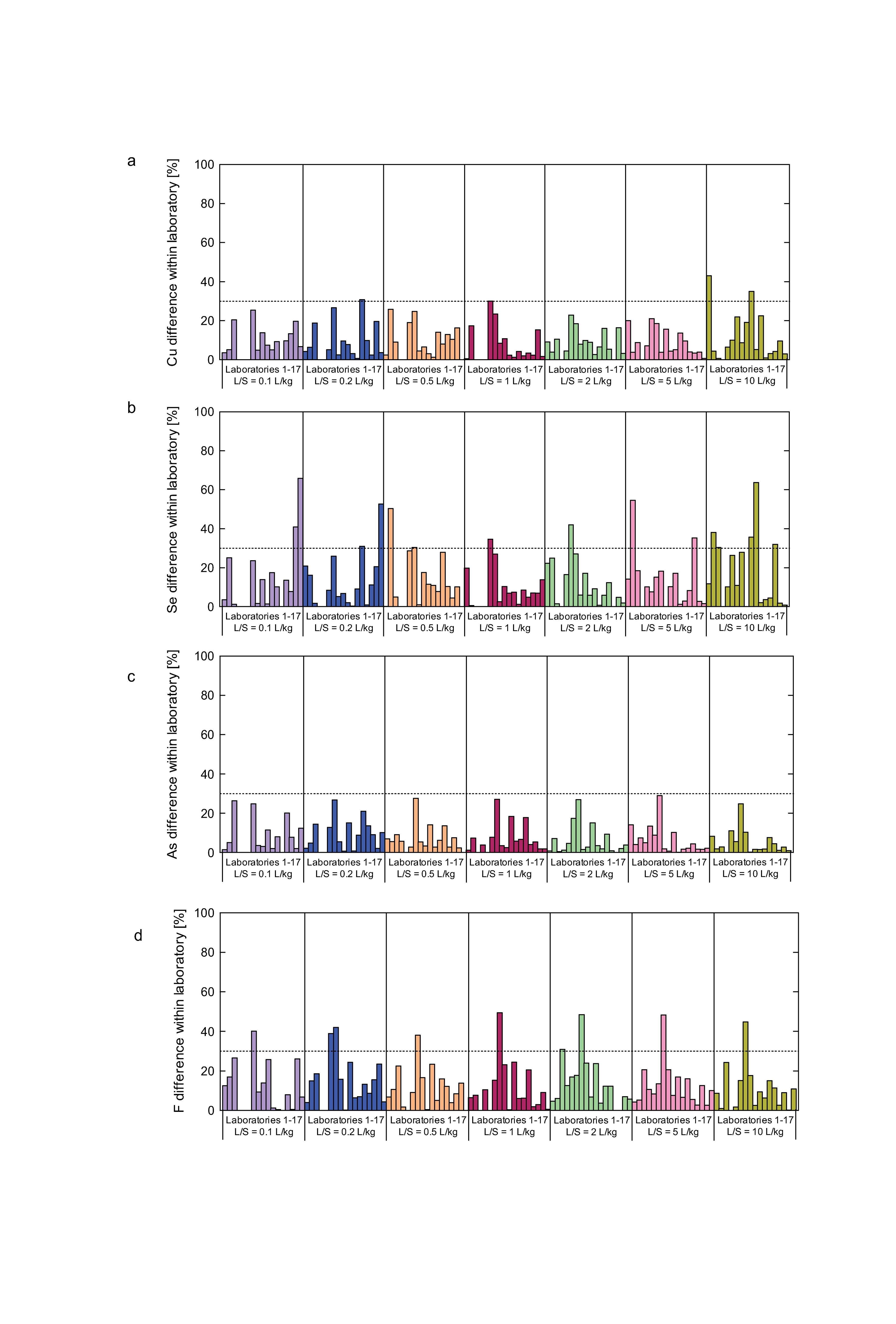

Supplement: S6 Fig — The “difference within laboratory” corresponds to the difference between cumulative releases obtained in the same laboratory, divided by their mean and expressed in terms of percentage. (TIF) [file pone.0178979.s006.tif]
