# Supplementary material for: Reproducibility of up-flow column percolation tests for contaminated soils
Source: PLoS One. 2017 Jun 5;12(6):e0178979. doi: 10.1371/journal.pone.0178979 (PMC5459554; doi:10.1371/journal.pone.0178979)
Supplement: S1 Table — (DOCX) [file pone.0178979.s007.docx]

**S1 Table. Effective data obtained from interlaboratory column tests for Soil I.**

| Parameter | Effective data/all data | |
| --- | --- | --- |
|  | Concentration | Cumulative release |
| Flow rate | 228/238 | NA |
| Liquid to solid ratio | 227/238 | NA |
| pH | 223/238 | NA |
| EC | 223/238 | NA |
| Cu | 214/238 | 214/238 |
| As | 223/238 | 223/238 |
| Se | 222/238 | 216/238 |
| Cl | 223/238 | 223/238 |
| Ca | 223/238 | 223/238 |
| F | 223/238 | 223/238 |

NA: not applicable
